# CRISPR-Cas9 and Its Bioinformatics Tools: A Systematic Review

**DOI:** 10.3390/cimb47050307

**Published:** 2025-04-27

**Authors:** Alicja Jasieniecka, Inês Domingues

**Affiliations:** 1 Polytechnic University of Coimbra, Rua da Misericórdia, Lagar dos Cortiços, S. Martinho do Bispo, 3045-093 Coimbra, Portugal; alicja.jasieniecka@onet.pl; 2 Medical Physics, Radiobiology and Radiological Protection Group, Research Centre of the Portuguese Institute of Oncology of Porto (CI-IPOP), 4200-072 Porto, Portugal

**Keywords:** CRISPR, bioinformatics, GMO tools

## Abstract

CRISPR-Cas9 has revolutionized genetic research with bioinformatics tools essential for tasks like guide RNA design, off-target prediction, and data analysis. This systematic review summarizes the functionality and key features of such tools. Studies published after 2012 were selected through searches in PubMed, Google Scholar, and other sources, with the final search conducted on 9 November 2024. Seven studies met the criteria, describing around 45 tools, including databases and functional programs. Tools like CRISPResso, CHOPCHOP, and Cas-OFFinder were commonly highlighted, with a major focus on single-guide RNA (sgRNA) design and optimization. Some tools provided specific solutions, while others offered broader functionality, but most lacked experimental validation. Several tools were developed by the authors of the studies, introducing potential bias. Findings highlight a need for integrated platforms that combine functionalities, reducing reliance on fragmented workflows. Current tools often address narrow tasks, complicating their practical application. Future development should focus on comprehensive, multitasking tools to improve accessibility and streamline research processes. Limitations include the descriptive nature of most studies, potential author bias, and challenges in comparing tools objectively. Nonetheless, this review underscores bioinformatics’ critical role in CRISPR research and emphasizes the need for innovative, standardized platforms. This study received no funding and was not registered.

## 1. Introduction

This paper presents a systematic review of CRISPR-Cas9 and the bioinformatics tools that support its usage. CRISPR-Cas9, a revolutionary gene modification technology, has transformed molecular biology and biomedical research since it was discovered in 2012 [1] by Emmanuelle Charpentier and Jennifer Doudna (this discovery landed them a Nobel prize in Chemistry in 2020 [2]). Enabling precise changes in genomic sequences, it paved the way for new ideas in medicine, agricultural improvements, and fundamental biological research.

CRISPR-Cas9 (sometimes called “Molecular scissors”) works as a precise gene-editing tool by using a guide RNA (gRNA) and the Cas9 enzyme (from the bacteria species called *Streptococcus pyogenes*). The process begins with the guide RNA, which is designed to match a specific DNA sequence in the edited genome. The Cas9 enzyme follows the guide RNA to this target sequence by binding to it through complementary base pairing.

Once the guide RNA brings Cas9 to the correct location in the DNA, the Cas9 enzyme cuts both strands of the DNA at the targeted site. This creates a double-strand break, which turns on the cell’s natural DNA repair mechanisms. The cell can repair the break in one of two ways: non-homologous end joining (NHEJ), which often results in small insertions or deletions that can disrupt gene function, or homology-directed repair (HDR), where a specific DNA template is provided to precisely alter the sequence [3].

This method allows for the targeted deletion, insertion, or modification of genes with high precision, making CRISPR-Cas9 a powerful tool for genetic research and potential therapeutic applications. The new technology is also much more affordable than previous gene-editing methods, rendering advanced genetic modifications accessible to a wider range of researchers and institutions, thereby accelerating innovation and discovery across various fields, including agriculture, medicine, and biotechnology.

The complexity, the sheer amount of genomic data, and the precision required in CRISPR-method genome editing have caused the rapid development of a wide range of bioinformatics tools, like CRISPRFinder or CRISPRtionary [4]. These tools are essential for designing CRISPR experiments, predicting off-target effects, analyzing data, and ensuring the accuracy and efficiency of the editing process. Given the broad and ever-expanding landscape of bioinformatics resources available for CRISPR-Cas9 applications, there is a critical need to systematically assess their utility, scope, and limitations.

The rationale for this review stems from the growing public and scientific interest in CRISPR-Cas9 in research and clinical settings, where the selection of correct bioinformatics tools can significantly impact experimental success and results. Existing literature offers scattered insights into individual tools, methods, and applications, but there is a lack of overall analysis that compares their performance, specificity, user-friendliness, and overall effectiveness in guiding CRISPR-based experiments. Furthermore, as CRISPR technology evolves, the bioinformatics tools and systems evolve as well. New algorithms, databases, and prediction models are continuously being developed, creating a dynamic field that requires ongoing evaluation, which individuals may find hard to keep up with [5].

This systematic review aims to address key questions surrounding CRISPR-Cas9 bioinformatics:

• Which bioinformatics tools are most commonly used, and for what purposes?
• What are the comparative strengths and limitations of these tools?
• How well do they perform in terms of accuracy, efficiency, and ease of use?
• What new tools are currently being developed, and what functions are still to be developed?

By critically examining the existing bioinformatics platforms available for CRISPR-Cas9, this review seeks to provide an updated and reliable reference for selecting appropriate tools for specific tasks, allowing for more effective genome editing projects.

## 2. Materials and Methods

This systematic review aimed to identify and analyze bioinformatics tools for CRISPR-Cas9, detailing their functions, accessibility, and impact on genome editing. Studies were primarily identified using Google Scholar and supplemented by the NIH’s National Library of Medicine, with occasional use of other platforms and educational videos from Polish YouTubers to access linked articles, with the final search conducted on 11 October 2024.

A wide range of search terms related to CRISPR and gene editing was used to locate studies, including “GMO”, “CRISPR”, “CRISPR-Cas9”, “genetic modification”, “gene editing”, “modifying genes”, “bioinformatic databases”, “bioinformatic tools for biologists”, and “informatics in GMO”. English- and Polish-language articles were included.

The article selection process involved abstract screening followed by a focused review of promising studies. The review prioritized widely used, accessible, and effective bioinformatics tools that streamline the CRISPR-Cas9 workflow, highlighting both established and newly developed options.

Bioinformatics tools were evaluated based on their role, accuracy, and ease of use in the CRISPR-Cas9 gene-editing process and organized by function, application, and type into categories, with data manually summarized from reputable, high-citation sources, prioritizing tool summaries over experimental findings and ensuring consistency by excluding low-quality studies.

The risk of bias was manually assessed based on study relevance, publication year, clarity of conclusions, and research methodology. The risk of reporting bias was assessed qualitatively, with studies excluded based on relevance, date, and quality.

Concerning the use of GenAI (gpt-3.5-turto), ChartGPT (gpt-4) was used mainly to find synonyms, check grammar, and perform other similar tasks. More details on the methods can be found in Appendix A.

## 3. Results

The included studies emphasize the pivotal role of bioinformatics tools in advancing CRISPR-Cas9 applications, including gRNA design, gene essentiality screening, and minimizing off-target effects. Tools such as MAGeCK, CERES, CRISPRFinder, and DeepCRISPR demonstrate utility in areas like off-target prediction, CRISPR array detection, and enhancing the accuracy of CRISPR screens. Databases like CRISPRdb and CRISPR-Casdb enable comprehensive storage and comparison of annotated CRISPR data, while tools like CRISPRDetect and CRISPRmap classify CRISPR systems and targets. Despite their value, the studies highlight ongoing challenges, such as predicting sgRNA efficiency and addressing genomic variations, which need further research. Risk of bias was generally low-to-moderate, though concerns about standardization and data transparency were noted. The evidence is moderately to highly certain, with consistent findings across studies, despite variability in tool design, experimental setups, and limited sample sizes. No formal statistical synthesis or sensitivity analyses were conducted, but tools like MAGeCK and CRISPRDetect showed robustness under diverse conditions.

### 3.1. Study Selection

Bias was minimized by focusing on relevance, quality, and CRISPR-Cas9 alignment. Pre-2012 studies were excluded, and those with significant conflicts of interest were omitted. Priority was given to newer, highly cited studies in reputable journals, with conflicts addressed by including both perspectives. Author credibility was evaluated based on expertise in CRISPR-Cas9 or bioinformatics.

#### 3.1.1. Initial Selection

The initial search in PubMed yielded over 2700 articles after using specific phrases like “CRISPR-Cas9 bioinformatics”, “CRISPR computer tools”, “Artificial Intelligence CRISPR”, and other similar terms, with the goal of identifying studies directly relevant to bioinformatics resources for CRISPR-Cas9. Filters were applied to restrict the results to studies published after 2012 in English or Polish, as CRISPR-Cas9 technology was first discovered in 2012, marking a clear starting point for this area of research. This choice was very important, as studies before this date might reference CRISPR more broadly—CRISPR elements themselves were known before 2012—but would not address the specific CRISPR-Cas9 gene-modifying technology, which is the main focus of this systematic review. So, excluding pre-2012 studies ensured the inclusion of only those articles relevant to this more recent and specific gene-editing technology.

The volume of results required a practical selection approach, as the review was conducted independently and without the help of automation tools. Consequently, the first 50 articles from PubMed were screened (since they were probably the most relevant and matched the most used phrases), of which 30 were deemed potentially relevant based on their titles and abstract review. During this phase, some studies were excluded due to limited access to full texts or the presence of significant conflicts of interest, but most of them were excluded because of a lack of relevant information for this review, resulting in a subset of 15 articles chosen for a more detailed examination.

From these 15 studies, 7 were ultimately chosen for inclusion as they presented detailed, relevant information about CRISPR-Cas9-specific bioinformatics tools, focusing on their applications, functionalities, or limitations. The remaining eight articles were excluded primarily for not providing specific information about bioinformatics tools; instead, they largely discussed CRISPR-Cas9 technology’s overall applications and predictions or detailed physical tools rather than the computational tools that are the focus of this review.

Additional records were identified by checking the references of the articles initially found (30 in total, with 21 finally included). After screening these references, those already included in the article were selected for direct citation, as they were relevant and frequently referenced in the chosen articles. This approach ensured the inclusion of pertinent sources directly linked to the specific article information.

**Figure 1:**
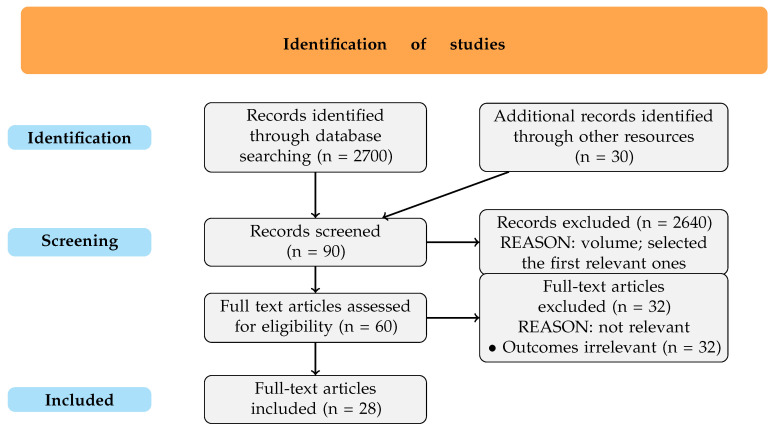
PRISMA-style flow diagram.

For enhancing transparency and clarity in reporting, a PRISMA-style flow diagram (Figure 1) is included to visually represent each stage of the study selection process, offering a concise overview of the steps from the initial article search through the final selection of studies included in this review. This diagram illustrates how the initial thousands of articles were systematically narrowed to a small and easy-to-follow amount.

#### 3.1.2. Minimizing Risk of Bias

To minimize the risk of bias, this study’s selection process was strict, focusing on article relevance, quality, and alignment with CRISPR-Cas9 bioinformatics tools. Studies published before 2012 were excluded to ensure that the findings aligned with CRISPR-Cas9 technology, discovered in that year. This distinction helped avoid research that might reference early CRISPR knowledge but lacked a clear focus on CRISPR-Cas9’s gene-editing applications. In addition, journals rooted in fields entirely unrelated to bioinformatics, genetics, molecular biology, computational biology, or computer science were not selected, as they would not align with the scientific rigor required for this review.

Studies were further assessed for conflicts of interest on a case-by-case basis, especially if the relevance of the article or study to the review’s objectives was high. In cases where conflicts of interest existed but were minor or involved a small proportion of researchers and/or authors, the study was generally included. However, if conflicts involved a large percentage of the authors or if the conflicts were considered substantial, the study was excluded. This selective approach allowed relevant research to be included while minimizing the potential bias from undue conflicts of interest, ensuring a high standard of scientific focus and quality.

To reconcile conflicting findings across studies, several criteria were employed. Studies that were more recent, published in high-impact journals, or had higher citation counts were often prioritized. Given CRISPR-Cas9 technology’s relatively recent development, newer studies were likely to provide more accurate information and reflect current technology standards. In cases where two studies met these requirements similarly, both were included to illustrate the conflict, with an indication that additional research may be required for clarification. For further context, popular science sources (like YouTube videos or scientific blogs and websites) were sometimes consulted when disagreements were found, though these sources were not given the same weight as primary scientific research.

Finally, authors’ backgrounds were occasionally reviewed to confirm expertise in CRISPR-Cas9 and bioinformatics fields. Authors with a consistent publication history in this area were assumed to have higher credibility than those new to the field, contributing an additional layer of reliability. To summarize, this risk-of-bias assessment applied rigorous standards, balancing the inclusion of highly relevant studies with quality indicators like citation counts, recency, and author credibility.

### 3.2. Study Characteristics

The included studies highlight the critical role of bioinformatics tools in optimizing CRISPR-Cas9 technology for applications like gRNA design, essential gene identification, and reducing off-target effects. Tools such as MAGeCK, CERES, and SSC improve the efficiency of CRISPR systems. While these studies provide valuable insights, they also note limitations, including challenges in predicting sgRNA efficiency, off-target effects, and genomic variations. These ongoing issues emphasize the need for continued research and development of more accurate bioinformatics tools.

Alkhnbashi et al. [4] present a comprehensive overview of the CRISPR-Cas systems and details the structural components of these systems, including a repeat-spacer array, a leader sequence, and various cas genes that encode proteins crucial for processing genetic information. CRISPR-Cas systems perform three essential functions: adaptation, involving the incorporation of foreign genetic material; the biogenesis of CRISPR RNAs (crRNAs) for guidance; and interference, focusing on degrading invading DNA or RNA. The article discusses various bioinformatics tools designed to predict the presence of these systems by identifying cas genes and CRISPR arrays, utilizing tailored approaches because of the unique features of these components. Several tools, such as CRISPRFinder, PILER-CR, and CRISPRDetect, are highlighted for their efficiency in identifying CRISPR arrays based on direct repeat (DR) sequences, local self-alignments, and regex searches, respectively. Moreover, the article explores databases like CRISPRdb and CRISPRCasdb, which provide extensive information on CRISPR arrays and Cas annotations, crucial for understanding the genomic context and evolutionary history of CRISPR systems.

The primary objective of Naeem and Alkhnbashi [6] is to review and evaluate bioinformatics tools designed to optimize CRISPR/Cas9 experiments, focusing on reducing off-target effects that can lead to unintended mutations. This review systematically analyzes literature on gRNA design, delivery methods, off-target detection techniques, and post-experimental analysis tools. Key bioinformatics tools discussed include CHOPCHOP, Cas-OFFinder, and CRISTA for optimizing gRNA sequences, as well as off-target detection methods such as MOFF, TIDE, and CRISPResso2. While the review synthesizes findings from multiple studies, it acknowledges limitations in detecting INDELs and genetic variability among organisms, putting in focus the need for tools that integrate this variability for better assessments. The study is highly relevant to the systematic review, as it encapsulates the current landscape of bioinformatics tools for CRISPR/Cas9 applications and contributes to understanding the effectiveness and challenges associated with CRISPR technologies.

Li et al. [7] develop and validate the MAGeCK algorithm for identifying essential genes from genome-scale CRISPR/Cas9 knockout screens. MAGeCK upgrades sensitivity and controls False Discovery Rates (FDRs) through a methodology that includes median-normalizing read counters and applying a negative binomial model. The study analyzes data from three distinct CRISPR/Cas9 knockout experiments and highlights MAGeCK’s superior performance compared with existing tools. The research significantly contributes to understanding bioinformatics tools in CRISPR-Cas9 applications, demonstrating MAGeCK’s effectiveness in enhancing the identification of essential genes and pathways.

Xu et al. [8] identify sequence features that enhance the efficiency of single-guide RNA (sgRNA) in CRISPR applications. The researchers developed Spacer Scoring for the CRISPR (SSC) software package (version SSC0.1), which analyzes genomic sequences to predict sgRNA efficiency based on specific nucleotide compositions. Key findings reveal that the Protospacer Adjacent Motif (PAM) and nucleotide compositions significantly influence sgRNA performance. While the study provides valuable insights into optimizing sgRNA design, it also notes limitations, as approximately 40% of inefficient sgRNAs remain unpredictable.

Meyers et al. [9] investigate the identification of essential genes for cancer cell proliferation using CRISPR-Cas9 technology. The primary bioinformatics tool introduced is CERES, designed to correct for the copy number effect in CRISPR-Cas9 screens. CERES enables unbiased interpretation of gene dependency and enhances the recall of essential genes necessary for cancer cell survival. The study employs robust data collection processes and validation methods, highlighting the critical role of bioinformatics tools in refining CRISPR-Cas9 essentiality screens. The findings contribute to understanding how computational corrections can mitigate the impact of genomic confounding factors, improving the accuracy of essential gene identification in cancer research.

The importance of effective sgRNA design for successful genetic manipulation is emphasized by Doench et al. [10]. Researchers delivered sgRNAs via lentiviral vectors into mouse and human cells and evaluated sgRNA efficacy across diverse gene targets. The study developed predictive models based on specific nucleotide preferences observed in active sgRNAs, optimizing sgRNA library design for improved success rates in gene editing. However, limitations include variability in sgRNA activity across different contexts, affecting the reliability of predictions regarding sgRNA efficacy.

Joung et al. [11] explore high-throughput genetic perturbation technologies for understanding gene function and epigenetic regulation. The primary objective is to show how the CRISPR-Cas9 system, particularly the use of dCas9 for transcriptional activation, can enhance our ability to manipulate gene expression effectively. By employing pooled sgRNA libraries for simultaneous gene perturbation, the study allows for loss-of-function (LOF) and gain-of-function analyses across various conditions and cell types. The research provides valuable insights into the application of CRISPR-Cas9 technology for genome-scale screening, contributing to the understanding of gene function and regulation. It aligns with the objectives of the systematic review by showcasing the advancements and effectiveness of bioinformatics tools in genetic research.

The following is a summary of the included articles:

- The articles collectively underscore the important role of bioinformatics tools in optimizing CRISPR-Cas technology for various applications, including gRNA design, essential gene identification, and off-target effect reduction.
- Each study presents unique methodologies and tools, such as MAGeCK, CERES, and SSC, enhancing the understanding of CRISPR systems and improving the efficiency of genetic manipulations.
- Limitations across the studies highlight ongoing challenges in accurately predicting sgRNA efficiency, off-target effects, and genomic variations, emphasizing the need for continued research and tool development in this evolving field.

### 3.3. Risk of Bias in Studies

Overall, the articles demonstrate a low-to-moderate risk of bias, primarily benefiting from reputable funding sources. While many use established methodologies, some show minor subjectivity due to a lack of standardized criteria or data transparency. Concerns about generalization arise from limited data sources and potential conflicts of interest. Despite these issues, most provide a solid foundation for their conclusions.

The work of Alkhnbashi et al. [4] shows a low-to-moderate risk of bias. It received funding from the DFG (SPP 2141 grants VO 1450/6-1 and BA 2168/23-1), an independent, non-commercial research organization, which minimizes bias from funding sources. Since the article focuses on summarizing available bioinformatics tools without direct testing, there is no bias in sample or data selection. However, tool comparisons lack standardized criteria, which could introduce minor subjectivity. Although some limitations are mentioned, such as availability and processing speed, other tools are not critically assessed, likely due to a genuine lack of documented limitations. This omission slightly affects transparency, though overall, the article maintains a low risk of bias due to its descriptive approach and neutral funding.

Naeem and Alkhnbashi [6] use both internal and external data sources, as a review of a range of bioinformatics tools specifically designed for sgRNA design and off-target detection is performed. This integration of data sources likely minimizes bias by encompassing a broader perspective on tool efficacy; however, because the study does not provide a detailed breakdown of individual data origins, there is a potential for minor bias due to limited transparency. As the article is primarily a review and not a primary research study, it does not include original experimental data, which reduces risks associated with data collection bias but also limits the depth of validation evidence for each bioinformatics tool. Funded by King Fahd University of Petroleum and Minerals, the study appears to carry a low risk of bias related to financial or institutional influence, as no direct affiliation with the tools assessed is evident. Overall, the absence of detailed primary data and sample information, combined with the broad review format, suggests a low risk of bias, though transparency in data origins could be improved.

The study by Li et al. [7] shows a moderate risk concerning source reliability, as it used a mix of open-access and restricted datasets, making most of its data reasonably accessible to researchers and students. For algorithm validation, the risk is moderate-to-high because the study developed a new tool but did not apply external validation or test on independent datasets, which may limit the generalization of its results. There is a possible risk associated with data limitations, as the completeness of the data and assumptions during analysis were not clearly specified. Finally, the funding and conflicts of interest are assessed to be at a low risk, with no competing interests declared by the authors and funding from reputable sources such as the NIH and the Dana–Farber Cancer Institute, suggesting minimal commercial influence.

A low risk of bias is present in [8], as it incorporates both well-known experimental techniques, such as Western blotting, and reliable databases. No significant data limitations were found, showing a comprehensive analysis. The study also includes validation through multiple datasets and experimental verification, further supporting its reliability. There is minimal risk of bias due to funding, as the project received support from reputable organizations like the NIH and NSF, with no direct corporate affiliations to the developed software, SSC. The software is openly available via SourceForge (https://sourceforge.net/, accessed on 24 April 2025), enhancing transparency.

The work of Meyers et al. [9] shows a low risk of bias because of its use of reputable data sources, including the Cancer Cell Line Encyclopedia (CCLE) and well-established algorithms such as PoolQ for data deconvolution and Bowtie for genome mapping. The incorporation of open-source software (Eigen 3.3 and ‘RccpEigen’ package) and the availability of code for replication further enhance the reliability of the findings. No significant data limitations were identified; the study’s thoroughness in data management and processing suggests it properly addressed potential issues that could affect results. However, the financial support from several grants and the Slim Initiative for Genomic Medicine, along with the involvement of author W. C. Hahn, who reported receiving a commercial research grant from Novartis and serving as a consultant for this company, introduces a moderate risk of bias, as such commercial interests may influence research focus or interpretation. Other authors disclosed no potential conflicts of interest.

A moderate risk of bias is found in [10]. A predictive model for sgRNA activity is developed using a logistic regression classifier trained on data from multiple mouse and human genes, employing reputable sequence features for activity predictions and cross-validating its model across different genes. But the reliance on data from a specific human melanoma cell line may limit the generalization of the findings. When extensive datasets were used, potential biases may arise from the specific characteristics of the genes selected for analysis and the limitations of the datasets used for validation, particularly in off-target predictions. The authors declared no competing financial interests, reducing the risk related to financial incentives, although funding from multiple NIH institutes may influence research directions. Notably, the model was validated using a substantial independent dataset of sgRNAs, enhancing the robustness of the findings, and the provision of a web tool for sgRNA scoring facilitates accessibility and potential reproducibility.

The study by Joung et al. [11] presents a moderate risk of bias for several reasons. It uses well-established genomic screening methods and the GeCKO v2 and SAM libraries, which are designed to minimize off-target activity and prioritize sgRNAs with fewer potential off-target sites. The scripts and libraries used are available from available sources, including Addgene and the Zhang laboratory. However, while the study outlines a good design for sgRNA libraries and screening analysis methods, it lacks mention of potential limitations in the datasets or biases introduced by the experimental design, such as the conditions under which the screening was conducted. Also, the authors acknowledge financial support from various institutions and disclose competing financial interests, particularly noting that F. Z. is a founder and advisor for Editas Medicine and Horizon Discovery. These relationships could influence the interpretation of the results and the emphasis placed on the findings related to the tools and methods discussed.

### 3.4. Results of Individual Studies

The CRISPR-Cas9 bioinformatics tools presented in Table 1, Table 2, Table 3, Table 4, Table 5, Table 6 and Table 7 cover a range of functions essential for gene-editing experiments, from CRISPR array detection to off-target effect prediction and gRNA design. Tools like CRISPRmap and CRISPRtarget aid in CRISPR system classification and target identification, while prediction tools such as CRISPRFinder and PILER-CR detect CRISPR arrays in genomes. Databases like CRISPRdb and CRISPR-Casdb store annotated CRISPR data for various organisms, facilitating comparison and analysis. Off-target detection tools, including MOFF and DeepCRISPR, enhance editing specificity, and gRNA design tools like Cas-OFFinder optimize on-target and off-target outcomes. Finally, tools like MAGeCK and CERES improve the accuracy of CRISPR screens, enabling better experimental design and gene analysis.

Repair Outcome Prediction Tools are bioinformatics resources that help anticipate DNA repair outcomes through non-homologous end joining (NHEJ), microhomology-mediated end joining (MMEJ), and homology-directed repair (HDR) pathways. These tools are primarily used to forecast the success of gene knock-out and knock-in strategies, aiding researchers in experimental design. Leveraging machine learning, they predict repair patterns based on the genetic context and repair pathway, providing insights tailored to various cell lines and organisms. This adaptability makes them versatile for applications across diverse genetic research settings.

CERES is a sophisticated tool designed to enhance the accuracy of gene dependency analysis from CRISPR-Cas9 essentiality screens, particularly by accounting for the antiproliferative effects unrelated to gene knockout [9]. By decoupling gene-dependent effects from those induced by DNA cleavage, CERES minimizes false positives, improving the reliability of cancer vulnerability assessments. Its large-scale application across 342 cancer cell lines from diverse lineages broadens the dataset, providing robust insights into gene essentiality. Although CERES offers high-quality predictions validated through ROC analysis and independent datasets, its complexity may demand considerable computational resources and high-quality input data for optimal results.

The bioinformatics tools in CRISPR-Cas9 research support crucial steps in designing, executing, and analyzing gene-editing experiments [10]. The sgRNA Target Site Identification Tool facilitates targeted gene editing by pinpointing sgRNA sites within the NGG PAM context in human and mouse genomes, incorporating exonic and intronic sequences to allow customized, species-specific sgRNA design. Meanwhile, the Data Processing Pipeline for Illumina Sequencing offers a streamlined workflow for measuring sgRNA abundance across various experimental conditions, employing normalization (e.g., “Reads per Million”) and stringent filtering to ensure dataset quality—although this can sometimes result in valuable sgRNAs being excluded from analysis. Enhancing precision further, the sgRNA Activity Predictive Model applies machine learning to evaluate sgRNA efficacy based on nucleotide features, using SVM and logistic regression techniques to generate robust predictions; however, this complexity demands substantial computational power and expertise.

For off-target assessment, the Off-Target Scoring Tool is essential, drawing on external databases to assess potential unintended effects of sgRNAs and enhance the safety of gene-editing experiments. While these tools are invaluable for gauging specificity, their predictions still often require experimental validation to confirm off-target risks. Finally, Data Normalization and Analysis Tools are instrumental in ensuring that pooled screening data are both standardized and insightful, using log-fold change calculations to evaluate sgRNA effectiveness while normalizing for variability in gene expression. This enhances the reliability of cross-comparisons but requires careful calibration of normalization parameters to prevent bias. Together, these tools provide a robust suite for managing the complexities inherent in CRISPR-Cas9 gene-editing projects, from initial target site identification to final data analysis.

Makarova’s method [12] classifies CRISPR-Cas systems into two classes, six types, and nineteen subtypes, based on the architecture of Cas loci and protein families. It achieves a 99.8% accuracy, misclassifying only 4 out of 1942 loci, making it highly reliable for large-scale genomic classification. However, it requires extensive computational resources and expertise, limiting its accessibility. In contrast, CRISPRmap [13] classifies CRISPR arrays based on direct repeats (DRs), identifying 33 structural motifs and 40 sequence families from over 3500 repeats. It is a user-friendly web-based tool, making it accessible for researchers without deep bioinformatics knowledge. While it focuses on repeat sequences rather than entire CRISPR-Cas systems, it is ideal for evolutionary studies and metagenomic data analysis. CASPERpam, used to predict PAM sequences, processes 720,391 spacers from prokaryotic genomes and identifies 26,364 hits (3.7%). It is efficient for large-scale data but shows variable accuracy depending on the CRISPR class, with 12 predicted PAMs showing 7 matches or strong correlations to experimental data. Its reliance on computational predictions requires experimental validation for more reliable results.

CRISPRdb [14], CRISPRCasdb, CRISPRone [15], and Anti-CRISPRdb [16] are key databases supporting CRISPR-Cas research, each with distinct focus areas and dataset sizes. CRISPRdb catalogs 870 CRISPR arrays from 232 archaeal and 8069 arrays from 6782 bacterial genomes, primarily offering CRISPR array information with monthly automatic updates and user-friendly features like BLAST-based private database comparison. CRISPRCasdb builds on this by integrating Cas protein annotations, covering a slightly broader dataset—240 archaeal and 9242 bacterial genomes—making it more suitable for functional studies involving both CRISPR arrays and Cas genes.

In contrast, CRISPRone prioritizes accuracy and large-scale analysis, identifying CRISPR-Cas systems in 11,102 complete and 21,186 draft genomes. It offers advanced tools like MetaCRT and HMMER for precise array and Cas protein prediction, and uniquely addresses false CRISPR arrays (mock CRISPRs), making it ideal for metagenomic and incomplete genome data. Anti-CRISPRdb is more specialized, focusing solely on anti-CRISPR (Acr) proteins. Though smaller (106 non-redundant sequences in 6 main and 23 sub-families), its manual curation supports high accuracy, and its search, screen, and download functions make it valuable for research on CRISPR inhibition.

CRISPRFinder [17] and CRISPRCasFinder [18] are widely used, detecting repeats 23–55 nt long with >80% similarity and discarding arrays if spacer similarity exceeds 60%. CRISPRCasFinder adds an evidence rating (levels 1–4) to assess prediction quality. Both tools are user-friendly, available via web and command line. PILER-CR uses self-alignment and graph-based repeat clustering, filtering repeats with <90% conservation. It is efficient but less intuitive for users. CRT, focused on low memory use, scans with a sliding window and uses exact string matching. It is Java-based, platform-independent, and offers both GUI and CLI, making it practical for resource-limited systems. CRISPRDetect uses regex and local alignment, refining arrays by extending repeats. It filters tandem repeats and is moderately efficient, but offers only command-line and web access. CRISPRdisco [19] combines Cas gene and CRISPR detection using MinCED and curated Cas profiles. It supports complete system classification but lacks a web interface.

For orientation prediction, CRISPRstrand [20] applies a graph-kernel machine learning model, reaching 0.95 AUC ROC (vs. 0.88 from older tools), improving repeat classification and downstream analysis. CRASS [21] and metaCRISPR [22] target unassembled metagenomic reads. CRASS achieves 0.89 spacer order sensitivity and 0.99 specificity, scales linearly with read count, and handles 76–2000 bp reads. metaCRISPR uses 13.6 GB RAM for 271M reads (150 bp), running in 108 minutes on four threads, offering clustering and contig assembly, suited for complex metagenomes. CRISPRleader [23] finds leader sequences using clustering and string kernels; it supports visualization and outputs in HTML and BED. CRISPRtionary [24], a web tool, compares CRISPR loci across strains and outputs binary spacer matrices for phylogenetics.

DeepCRISPR uses deep learning to predict on- and off-target effects with improved accuracy, while MOFF incorporates mismatch tolerance and epigenetics, outperforming previous models, though exact accuracy numbers aren’t provided. In unbiased detection, GUIDE-seq is favored for its low cost, low false-positive rate, and broad applicability. GUIDE-Tag improves this with Tn5 tagging to reduce PCR bias, achieving sensitivity for off-target effects at ≥0.2% editing efficiency. PEM-Seq, combined with LAM-HTGTS, is highly sensitive for detecting genomic translocations. For prime editing, PEAC-Seq and TAPE-Seq [25] offer genome-wide detection, with TAPE-Seq providing live-cell analysis and both on- and off-target activity detection.

Guide RNA (gRNA) design tools optimize CRISPR/Cas9 efficiency and specificity. CHOPCHOP [26] is an early tool supporting over 100 organisms but lacks advanced features. Cas-OFFinder, integrated into CRISPR RNGE, uses machine learning for better off-target detection. CRISTA supports 100+ organisms but is limited to spCas9, while GuideScan focuses on mouse and human genomes, enhancing precision. CRISPRDo is versatile, supporting both Cas9 and Cpf1, and is effective for zebrafish, mice, and humans, incorporating epigenetic factors for better accuracy. sgRNACas9 targets the mouse genome and quantifies off-target effects. For plants, CRISPR-P is popular, while PhytoCRISPR and CRISPRz focus on plant genome editing. CRISPOR is the most versatile, supporting 30+ Cas variants and providing detailed off-target analysis. Png Designer specializes in base and prime editing. Tools like CRISPOR and CRISPRDo excel at off-target prediction. Numerical data show that truncating gRNA from 20 to 17 nucleotides can reduce off-target effects by 500-fold, and the optimal GC content for gRNAs is 40–60%. Overall, CRISPOR and CRISPRDo are highly versatile and accurate, while others like CRISTA and GuideScan are specialized but effective within specific domains.

Cas9-based GeCKO v2 libraries are more accurate and efficient than shRNA [27]. GeCKO screens target 19,050 human or 20,611 mouse genes with six sgRNAs per gene and also include non-targeting controls. These libraries identify more lethal genes, with lower false negative rates compared with shRNA. GeCKO’s ability to pinpoint essential genes is particularly beneficial for loss-of-function (LOF) screening, where complete gene inactivation is crucial. In contrast, shRNA often fails to achieve full gene knockdown, leading to less reliable results. Despite its advantages, Cas9 has limitations. In cancer genomes, Cas9-induced double-strand breaks (DSBs) can cause false positives due to gene-independent DNA damage [28]. Also, targeting the 5’ exons may produce in-frame variants that retain partial gene functionality, masking true genetic dependencies. For gene activation, the SAM (Synergistic Activation Mediator) library targets the 200 bp region upstream of the transcriptional start site of 23,430 human or 23,439 mouse genes, with three sgRNAs per isoform. SAM requires additional effectors in a two- or three-vector system for activation, allowing precise transcriptional control. GeCKO and SAM libraries prioritize sgRNAs with minimal off-target effects, optimizing the specificity and efficiency of the screening process. Custom sgRNA libraries can be designed using a Python script 2.7 [11] to minimize off-target activity, which is crucial for reliable results.

### 3.5. Results of Syntheses

This synthesis analyzes various bioinformatics tools for CRISPR-Cas9 applications, focusing on tools for CRISPR array detection, Cas gene identification, PAM prediction, off-target prediction, and gene essentiality screening. The studies highlight tools like CRISPRFinder, MAGeCK, and CRISPRdb, which employ diverse methods such as regex searches, machine learning, and graph-based analysis. No formal statistical synthesis was performed, but variability in tool performance across experimental conditions was observed, with tools like MAGeCK and CRISPRDetect showing robustness in different contexts. Sources of heterogeneity included differences in tool design and experimental setups. While no sensitivity analyses were conducted, variations in tool design were noted as affecting result robustness. Reporting biases were not evident, and the certainty of evidence is considered moderate-to-high, with consistent findings across studies despite some limitations like narrow sample sizes and potential biases in self-reported tool comparisons.

#### 3.5.1. Summary of Study Characteristics and Risk of Bias

The synthesis focuses on the analysis of various bioinformatics tools for CRISPR-Cas9 applications, including tools for CRISPR array detection, Cas gene identification, PAM prediction, off-target prediction, and gene essentiality screening. Key tools and databases discussed include CRISPRFinder, CRISPRCasFinder, PILER-CR, CRISPRDetect, CRISPRdisco, and others. Each tool employs diverse methods such as regex searches, graph-based analysis, and machine learning to optimize CRISPR detection and analysis across genomes. The studies collectively reflect a range of methods to improve CRISPR/Cas9 specificity and efficiency. For instance, CRISPRdb and CRISPRCasdb focus on CRISPR loci and anti-CRISPR protein data, while tools like MAGeCK are focused on identifying essential genes in CRISPR screens. No formal statistical synthesis was conducted across studies due to the descriptive nature of the tools, but each study emphasizes the strengths and intended applications of these tools in diverse genomic contexts. The risk of bias across the studies is generally low-to-moderate, but variability exists due to the use of different data sources and experimental conditions.

#### 3.5.2. Statistical Syntheses Results

No formal meta-analysis or statistical synthesis was performed across the studies, as most tools were evaluated descriptively rather than compared quantitatively. However, the studies implicitly address variability in tool performance across different experimental conditions. For instance, tools like MAGeCK were tested in different cell types, showing their robustness in identifying essential genes. Similarly, tools like CRISPRDetect and CRISPRdisco were highlighted for their refinement in CRISPR array detection, though they vary in their precision based on the tools’ designs and the complexity of the genomic data used. The direction of effects varies depending on the specific context, but tools designed for specific tasks (e.g., off-target prediction or gene essentiality screens) show improvements in CRISPR/Cas9 specificity and effectiveness. The studies suggest that combining different tools is often necessary for comprehensive CRISPR analysis.

#### 3.5.3. Investigations of Causes of Heterogeneity Among Study Results

The studies identified several sources of heterogeneity in the results. For example, the variation in CRISPR array detection methods, such as those used by CRISPRFinder and CRISPRCasFinder, shows how differences in approach (e.g., regex-based vs. graph-based analysis) can affect detection sensitivity. Similarly, the use of machine learning for off-target prediction in tools like E-CRISP and CHOPCHOP contrasts with sequence alignment-based methods, highlighting the complexity of achieving standardized results across different organisms and experimental setups. Factors like the delivery methods for CRISPR/Cas9 (e.g., ribonucleoprotein versus plasmid-based delivery) and differences in genome complexity between species also contribute to the observed heterogeneity in tool performance.

#### 3.5.4. Sensitivity Analyses

No explicit sensitivity analyses were conducted in the studies, but variations in tool design were discussed as factors affecting the robustness of results. For instance, tools like CASPERpam require large input datasets for accurate PAM prediction, while tools like SSC for sgRNA efficiency emphasize sequence features that contribute to off-target effects. Additionally, studies discussed specific optimization strategies to improve tool sensitivity, such as varying Cas-to-gRNA ratios and truncating gRNAs. While no formal sensitivity analysis was performed, these modifications suggest efforts to refine the robustness of CRISPR tool results across different contexts. Tools like MAGeCK demonstrated robustness even with reduced sequencing depth or fewer sgRNAs, showing their reliability under variable experimental conditions.

#### 3.5.5. Reporting Biases

There were no apparent issues with missing results or unpublished data that could have affected the conclusions drawn in this review. Although there is no direct evidence of reporting bias (e.g., selective outcome reporting), it is possible that some missing data may have been overlooked due to the nature of the review process. The studies reviewed were primarily from reputable sources, and the risk of bias was generally low-to-moderate, as discussed in the overall risk of bias section. None of the studies involved specific tools for detecting reporting bias, such as funnel plots, as the analysis was conducted manually. While the articles often cited established methodologies and had funding from reputable sources, a few studies exhibited minor subjectivity. This subjectivity arose due to the lack of standardized criteria or transparency in some of the data presented, which could have contributed to selective reporting in those cases. However, despite these concerns, the studies provided a solid foundation for the conclusions.

#### 3.5.6. Certainty of Evidence

The certainty of evidence in this review is considered moderate-to-high. The included studies were drawn from respected databases such as NCBI and demonstrated low-to-moderate risk of bias. Most of the studies were professionally written, well cited, and showed reliable study design and results. Additionally, conflicts of interest were generally disclosed and did not appear to significantly influence the conclusions of the studies. Although no specific frameworks for assessing the certainty of evidence were used, the review found the evidence to be trustworthy due to the quality of the articles. However, there are a few limitations that may affect the certainty of the evidence. These include the narrow scope of some studies (e.g., limited sample sizes, testing on specific cell types or organisms) and the possibility of bias in articles authored by creators of the tools being discussed (e.g., MAGeCK and CERES), which may have led to favorable comparisons of their own tools against others. Despite these limitations, the evidence was generally consistent, with similar findings reported across multiple studies regarding the tools discussed. While some tools were only mentioned in one article, those mentioned in more than one showed consistent data. Given the specific nature of the tools reviewed, smaller sample sizes were expected and did not seem to significantly affect the results. Overall, the certainty of the evidence is supported by the credibility of the sources and the consistency of findings across the studies.

## 4. Discussion

This review synthesizes bioinformatics tools for CRISPR-Cas9 applications, highlighting widely used tools like CRISPRdb, MAGeCK, and CHOPCHOP, which advance gene editing and off-target prediction. While these tools reflect significant progress in CRISPR technology, limitations include a lack of empirical evaluations, detailed performance data, and variability in experimental designs. The manual selection process of studies also introduced potential biases. The review suggests that more integrated platforms combining multiple CRISPR functions could improve tool efficiency, while emphasizing the need for stringent regulation of genetic data. Future research should focus on developing more versatile, AI-driven, user-friendly tools for broader CRISPR applications.

### 4.1. General Interpretation of the Results

This review synthesized the bioinformatics tools developed for CRISPR-Cas9 applications, including tools for CRISPR array detection, PAM prediction, off-target prediction, and gene essentiality screening. Tools such as CRISPRdb, CRISPRFinder, CRISPRCas-Finder, CHOPCHOP, and CRISPOR are frequently mentioned across the literature, suggesting their wide usage and significance in advancing CRISPR-Cas9 technology. For example, MAGeCK and CERES are crucial for gene essentiality screening, while E-CRISP and CHOPCHOP are valued for their refinement of off-target prediction. The development of these tools reflects the rapid evolution of CRISPR technology, with an ongoing focus on optimizing sgRNA design and improving accuracy in genome-wide predictions. Despite significant advancements, CRISPR-Cas9 technology remains relatively young, with continued research needed to optimize the existing tools for broader applications.

### 4.2. Limitations of the Evidence

While the studies included in this review provide valuable insight into various CRISPR bioinformatics tools, several limitations were noted. Many studies primarily focused on describing the tools rather than evaluating them empirically. This limited the ability to perform a formal statistical synthesis or meta-analysis. Furthermore, the studies lacked detailed performance data, such as accuracy or processing time, which would have been helpful for comparing the tools. The tools reviewed are often highly specialized, focusing on narrow aspects of CRISPR-Cas9, such as specific gene-editing tasks or off-target prediction, making it difficult to draw direct comparisons across different tools. Additionally, there was considerable heterogeneity in experimental designs, genome types, and conditions across the studies, which affected the consistency of results.

### 4.3. Limitations of the Review Processes

A significant limitation in the review process was the manual screening and selection of articles. While over 2500 articles were identified, only 50 abstracts were reviewed in detail, and around 30 full-text articles were read, with fewer than 10 selected for inclusion. The absence of automated tools to assist in the analysis of articles may have introduced potential biases in the selection process, as articles focusing on bioinformatics tools were prioritized over those discussing general CRISPR applications without specific references to the tools used. This limitation may have resulted in the exclusion of relevant data. Furthermore, the studies included in the review varied in focus, with many addressing the application of CRISPR-Cas9 in specific experimental contexts rather than the tools themselves, leading to an incomplete understanding of the full landscape of available bioinformatics tools.

### 4.4. Implications for Practice, Policy, and Future Research

AI and machine learning are transforming CRISPR bioinformatics, enhancing precision and minimizing off-target effects. Tools like CRISPRater, CRISPRScan, and DeepCRISPR are already improving the accuracy of on- and off-target predictions, which are critical for advancing CRISPR in personalized medicine and cancer research. For practitioners, adopting these tools is essential to optimize CRISPR applications in clinical settings. However, policies must ensure that these models are transparent, interpretable, and rigorously validated to facilitate their safe use in clinical environments [27,28].

Fom a policy perspective, the regulation of bioinformatics tools and databases is critical, especially as many of these tools handle sensitive genetic data. Given the growing use of genomic data in personalized medicine and research, stringent regulations should be implemented to ensure the secure handling and disposal of genetic information. The possibility of misuse, such as genetic discrimination or bioterrorism, underscores the need for careful oversight of genetic databases and related technologies. Future research should focus on addressing data limitations, particularly by incorporating multiomics and epigenetic data to enhance the performance of bioinformatics models. Cross-species prediction models will also be crucial for translational research, allowing CRISPR therapies to be more broadly applicable. Additionally, improving training data diversity and addressing class imbalance through techniques like data augmentation will be key to refining off-target predictions.

Further development of bioinformatics tools should aim to cover a wider range of CRISPR-Cas9 applications. This includes integrating AI-driven predictions for gene editing, developing more user-friendly platforms that combine various stages of the CRISPR process, and enhancing the accessibility of these tools. Open-source platforms incorporating the latest bioinformatics advancements will foster collaboration and benefit the global research community. Finally, while the tools discussed in this review provide significant advancements in CRISPR research, their specialized nature suggests a need for more comprehensive platforms that integrate multiple functions, such as off-target prediction, PAM identification, and sgRNA design. Such tools could simplify the user experience and improve the overall efficiency of CRISPR-Cas9 applications. These innovations are poised to significantly improve CRISPR-based applications, but ongoing efforts to improve data quality, model transparency, and resource accessibility are essential for their successful integration into clinical practice.

## 5. Other Information

This review was not registered, and no formal protocol was prepared. The review process was loosely planned, with articles and notes organized in Microsoft Word. No financial support was received, and the author accessed articles through Europe PMC using a university account. There are no competing interests. Data were manually summarized and organized without standardized forms or analytic software. While no quantitative analyses were performed, all directly cited sources are included in the references, and general background knowledge from pop science resources informed the review. Data are available upon reasonable request.

## Figures and Tables

**Table 1:** CRISPR-Cas Bioinformatics Tools [4].

Tool Name	Purpose	Key Features
CRISPRmap	Classification of CRISPR-Cas systems based on direct repeat (DR) sequence and RNA secondary structure conservation	Clusters CRISPR repeats into families and identifies conserved structural motifs; Provides comprehensive visual maps and clustering trees of DRs; useful for studying CRISPR-Cas system evolution, with applications in haloarchaeal and human gut microbiome research
CASPERpam	Predicts Protospacer Adjacent Motifs (PAMs) critical for targeting efficiency	Uses BLASTn to detect protospacer sequences and scores them for PAM prediction. Limitations: Initial predictions require further validation, as the method is dependent on protospacer matches
CRISPRtarget	General-purpose target prediction tool for identifying potential CRISPR targets in newly identified CRISPR systems	BLAST-based similarity search with options for additional filtering by PAM or handle lengths. Provides a flexible approach for target identification across custom and public databases
E-CRISP, CHOPCHOP, CRISPR-ERA, CRISPOR, GuideScan	Tools for designing specific gRNAs for CRISPR-Cas genome editing, focused on high on-target efficiency and low off-target activity	Prioritize target specificity and efficiency based on empirically derived scoring rules; Comprehensive off-target prediction using read mapping tools like BWA and Bowtie; Provide web interfaces and options for local installation to facilitate large-scale applications.

**Table 2:** CRISPR Databases [4].

Name	Purpose	Data Coverage	Features
CRISPRdb	First public database for CRISPR arrays in archaeal and bacterial genomes	870 CRISPR arrays from 232 archaeal genomes and 8069 from 6782 bacterial genomes	Monthly updates from NCBI Reference Sequence Database; allows private database creation and BLAST comparison with public data
CRISPR Casdb	Database for CRISPR arrays and Cas annotations, complementary to CRISPRdb	Includes data from 240 archaeal and 9242 bacterial genomes	Integrates with CRISPRCasFinder for CRISPR-Cas system prediction and offers extensive genomic annotation
CRISPRone	CRISPR-Cas identification with a focus on distinguishing true and mock (false) CRISPR arrays	Covers 11,102 complete genomes and 21,186 draft genomes	Uses MetaCRT for CRISPR array prediction and HMMER for Cas protein identification; predicts anti-repeat sequences, crucial for tracrRNA formation
Anti-CRISPRdb	Database for anti-CRISPR (Acr) proteins	Categorizes 106 proteins into 6 families and 23 sub-families	Provides tools for browsing, searching, downloading, and sharing data on Acr proteins. The only public database focused on anti-CRISPR proteins

**Table 3:** Prediction of CRISPR-Cas9 systems [4].

Tool Name	Purpose	Key Features	Availability
CRISPRFinder and CRISPRCasFinder	Identification of CRISPR arrays by detecting repetitive sequences	Use enhanced suffix arrays (via Vmatch) to efficiently locate repeats; offer evidence-level rating system (Levels 1–4) based on repeat number, conservation, and spacer identity	Command line (Unix-based), web server available
PILER-CR	Identifies CRISPR repeats using local self-alignment to build connectivity graphs	Constructs “piles” of repetitive elements and refines them into CRISPR array candidates; merges arrays with high similarity in repeat sequences	Stand-alone tool for Unix-based systems
CRISPR Recognition Tool	Memory-efficient identification of CRISPR arrays, with a focus on repetitive sequences	Sliding window search and user-defined mismatch allowance; Java implementation allows cross-platform functionality	Command line and GUI options available
CRISPRDetect	CRISPR array detection with a structured five-step approach	Filters candidates by tandem repeat similarity and extends sequences; offers local alignment to locate missing repeats	Command line for Linux, web server available
CRISPRdisco	Combined prediction of CRISPR arrays and cas genes	Uses BLAST-based search for cas genes; CRISPR array detection with MinCED and classification based on evidence	Local installation only (no web server)
CRISPRstrand	Determines CRISPR array orientation for functional analysis	Graph-kernel machine learning improves accuracy of orientation prediction; achieves AUC ROC of 0.95	Integrated into the CRISPRmap web server
CRASS	CRISPR-Cas detection in unassembled metagenomic reads	Graph-based approach reconstructs spacer arrangement; high sensitivity and specificity for short reads (76–2000 bp)	Command line, Unix-based, primarily optimized for Illumina reads
metaCRISPR	Identifies and assembles CRISPR arrays from unassembled metagenomic reads	Uses DR identification and read clustering for assembling arrays; integrates with Bowtie and Genometools	Command line for Unix systems, requires additional software
CRISPRleader	Detects CRISPR leader sequences for adaptation phase analysis	Identifies leader boundaries using string kernels for conserved motifs; distinguishes arrays in archaea and bacteria	Python-based, outputs CRISPR annotation in BED and HTML formats
CRISPRtionary	Annotates and compares spacer sequences for phylogenetic analysis	Creates spacer dictionaries and binary files to visualize strain spacer compositions	Web-based, requires FASTA input files

**Table 4:** Off-target detection methods [6].

Tool Name	Purpose	Key Feature	Scope
MOFF	Off-target detection for CRISPR-Cas9 to enhance editing specificity	Considers mismatch tolerance and epigenetic effects	Broadly applicable, CRISPR-Cas9
DeepCRISPR	Detects both on-target and off-target effects to improve editing accuracy	Uses deep learning for high precision	Multiorganism applications
PEM-Seq	Detects off-target genomic translocations at a high sensitivity	Effective in capturing translocations	Genomic-level applications, unspecified organisms
Guide-Tag	Identifies large deletions and translocations to assess genetic stability	Effective at low editing efficiencies	CRISPR-edited genomes, general use
PEAC-Seq	Detects off-target effects and genotoxicity in prime-edited cells	Effective in vivo and in vitro	Prime-edited cells, general use
TAPE-Seq	Monitors off-target effects in live cells, specifically for prime editing	Analyzes on-target and off-target activities	Prime editing in live cells

**Table 5:** Guide RNA (gRNA) Design Tools [6].

Tool Name	Purpose	Key Features	Scope
Cas-OFFinder	Efficient gRNA design with detection of on-target and off-target effects	Machine learning-based off-target detection	Broadly applicable, multiorganism
CRISTA	gRNA design with quantified on-target and off-target accuracy	Specific to spCas9	Supports multiple organisms
CRISPRDo	Enables simultaneous detection of on-target and off-target effects in gRNA design	Applicable to various model organisms	Zebrafish, mice, humans, worms
sgRNACas9	Specialized for off-target quantification in spCas9 gRNA design	Analyzes structural and sequence factors	Mouse genome
EupaDGT	Tailored gRNA design for genome editing in eukaryotic pathogens	Supports multiple Cas proteins	Eukaryotic pathogens
WU-CRISPR	Optimized human and mouse gRNA design using spCas9	Machine-learning-based design for spCas9	Human and mouse genomes
CRISPR-P	Specialized gRNA design for plant genomes and crop editing	Adapted for plant and crop genome editing	Plants and crops
CRISPRz	Database-trained gRNA design tool for plant-specific genome edits	Uses a large spCas9 database	Plant genomes
PhytoCRISPR	gRNA design for plant genome editing, supports spCas9 and Cas12	Compatible with two nuclease types	Plant genomes

**Table 6:** Analytical Tools for CRISPR-Cas9 Screen Data [7].

Name	Purpose	Key Feature	Pros/Cons
MAGeCK	Prioritizes sgRNAs, genes, and pathways in CRISPR/Cas9 knockout screens	Identifies essential genes through simultaneous positive and negative selection	**Pros:** High sensitivity and specificity; manages read count variability effectively **Cons:** Requires high-throughput sequencing data, which can be resource-intensive
edgeR	RNA-seq differential expression analysis and sgRNA significance in CRISPR screens	Models read count variability with a negative binomial distribution	**Pros:** Well-documented and widely used; efficient for high variance data. **Cons:** Less effective for CRISPR applications compared with MAGeCK, particularly at low read counts
DESeq	Analyzes RNA-seq data, adapted for sgRNA significance in CRISPR screens	Employs a negative binomial model for variance estimation	**Pros:** Robust variance modeling for RNA-seq; free and open-source **Cons:** Variable performance with CRISPR data compared with MAGeCK
baySeq	Detects significant changes in RNA-seq, applicable to CRISPR data	Uses an empirical Bayes approach for posterior probability estimation	**Pros:** Offers refined significance testing; suitable for complex experimental designs **Cons:** Computationally intensive; not fully optimized for CRISPR applications
NBPSeq	Identifies differentially expressed genes or sgRNAs in CRISPR screens	Applies a negative binomial distribution for assessing count data	**Pros:** Effective for over-dispersed high-throughput sequencing data **Cons:** Primarily designed for RNA-seq, may not fully capture CRISPR nuances
RIGER	Ranks genes based on functional importance, adapted from siRNA screens	Uses signal-to-noise ratios for gene ranking	**Pros:** Straightforward ranking mechanism for essential genes; useful in positive selection contexts **Cons:** Lower sensitivity in CRISPR applications; may miss some essential genes
RSA	Ranks genes based on redundancy in siRNA activity, adapted for CRISPR screens	Focuses on fold-change between treatment and control to identify active genes	**Pros:** Highlights consistently active genes effectively; useful for positive selection **Cons:** Prone to bias towards sgRNAs with fewer reads; higher false positive rates

**Table 7:** CRISPR Screening Tools and Methods [11].

Name	Purpose	Key Features	Pros/Cons
GeCKO	High-throughput LOF screening to identify essential genes	Targets over 19,000 human genes with 6 sgRNAs each; available in single and dual-vector formats	High consistency and lower false negatives compared with shRNA but may produce false positives due to DNA damage
SAM	Transcriptional activation screening	Targets 23,000+ coding isoforms with 3 sgRNAs each, used with additional effectors in dual or triple-vector formats	Focuses on minimizing off-target activity for reliability, though its complexity may require optimization
sgRNA Library Construction Tools	Design and construct custom sgRNA libraries for targeted experiments	Python scripts for generating sgRNAs with minimized off-target effects	Highly adaptable for diverse applications but requires computational skills to utilize effectively
Screening Analysis Methods	Analyze screening results to identify enriched or depleted sgRNAs and genes	Various methods (RIGER, RSA, MAGeCK, STARS) with distinct statistical approaches	Reduces false positives with robust analysis, but can be complex and demanding in terms of statistical interpretation

## Data Availability

No standardized data collection forms were used. Microsoft Word was used for organizing notes and summarizing articles informally. Extracted data were summarized and organized manually; these data are available upon reasonable request. The review was conducted impartially due to the difficulty of organizing large amounts of data manually. No quantitative analyses were performed in this review. No analytic code or software was used; all organization and synthesis were conducted manually. All sources directly cited in the review are included in the references. General background knowledge was gained from pop science videos, documentaries, books, and journals; these sources provided foundational understanding but were not directly cited.

## Appendix A

In this systematic review, the goal was to identify and analyze bioinformatics tools relevant to CRISPR-Cas9, focusing on their functions, accessibility, and impact on genome editing. Here, the methods that were used to select, screen, and analyze these studies are outlined.

### Appendix A.1. Eligibility Criteria

All bioinformatics tools that are useful for CRISPR applications were included, with a focus on those developed after 2012, the year CRISPR-Cas9 technology was discovered, which ensured relevance, although some older works were taken into account as well. Articles are only in English, since this language is widely understood within the scientific community, although Polish articles were also considered, given the first author’s fluency in this language. While the main focus was on bioinformatics tools, a small number of articles for general information on CRISPR technology and its applications were also reviewed.

### Appendix A.2. Information Sources

Studies were identified using mostly Google Scholar, which is the perfect tool for searching scientific articles, journals, or books, and occasionally using regular Google searches as well. The NIH’s National Library of Medicine is a website where the most-read articles are displayed, while websites like ScienceDirect, Web of Science, or Research Gate are used less frequently. In addition to these academic resources, educational videos from creators like Kasia Gandor and Naukowy Bełkot (Polish YouTubers who focus on biological sciences) were also consulted in order to access the links to articles included in their video descriptions, which allowed faster access to them. The last search for relevant studies was conducted on 11 October 2024.

### Appendix A.3. Search Strategy

A variety of keywords and phrases to locate studies were used, including “GMO”, “CRISPR”, “CRISPR-Cas9”, “genetic modification”, “gene editing”, “modifying genes”, “bioinformatic databases”, “bioinformatic tools for biologists”, and “informatics in GMO”. These terms were also combined for wider results. Articles published after 2012 were prioritized, with the majority of the studies published in the last decade. Entering keywords in English ensured that the results were in this language and did not make it necessary to use additional filters.

### Appendix A.4. Selection Process

The article selection process began by reading the abstract of each study. If the abstract suggested that the article might be useful for the research, the entire article was reviewed, focusing on paragraph titles, headings, and words in bold. Given the volume of articles, a fast, targeted screening method was used.

### Appendix A.5. Data Collection Process

The main goal of the search was to collect detailed information on bioinformatics tools used in CRISPR-Cas9 gene editing. The names of tools were looked for in websites, databases, downloadable applications, and with Artificial Intelligence (AI) models, along with descriptions of their functions. Specifically, the following information was recorded: each tool’s role in the CRISPR workflow, whether it simplified or accelerated specific tasks, its accessibility (free access, restricted to academic organizations, or pay-walled), its release date (the older tools may be less useful or not compatible with new ones), and any similar or complementary tools. Information on tools that are still being developed or gaps that need to be filled was additionally checked. Data were collected independently without the use of automation tools.

### Appendix A.6. Data Items

Given the large number of bioinformatics tools available, the most relevant and widely used ones were included for this review. Tools with the most comprehensive functionalities, ease of use, accessibility, and the ability to make CRISPR-Cas9 workflow easier were focused on. The review highlights tools that are commonly used, newly developed, or particularly effective in supporting genome editing processes.

### Appendix A.7. Study Risk of Bias Assessment

The risk of bias was assessed manually, without help from other researchers or automation tools. Each study’s relevance to the document focus was considered the main factor, along with the publication year (newer studies were preferred for their more up-to-date information). Additional factors, such as the clarity of conclusions and proper research methods, were also taken into account.

### Appendix A.8. Effect Measures

For this systematic review, no formal quantitative effect measures like risk ratios or mean differences were used, as the focus was on evaluating bioinformatics tools rather than clinical outcomes or continuous data. Instead, each bioinformatics tool was checked based on its specific outcomes, such as its role in the CRISPR-Cas9 gene-editing process and accuracy, as well as ease of use.

### Appendix A.9. Synthesis Methods

Studies and bioinformatics tools were organized by their functions and applications within the CRISPR-Cas9 process, categorizing them by roles (e.g., guide RNA design, protein structure prediction) and type (e.g., databases, target identification, AI prediction tools) for easier access to relevant information.

Data were compiled in Microsoft Word, where important parts from each study or website were pasted alongside their links and study names, and then structured into the categories mentioned in the previous paragraph. Data summarization was performed manually, without any special software, based on personal analysis and critical thinking. Summarizing existing tools was prioritized over experimental findings, emphasizing studies from reputable journals with higher citations and more recent publication dates in cases of conflicting information, which additionally reduced heterogeneity. Data from similar-quality studies were also included to present a comprehensive overview. Although no formal sensitivity analyses were performed, the integrity of the results was kept thanks to excluding low-quality studies that conflicted with the majority.

The main challenge in preparing the data was that the included studies largely provided lists or comparisons of bioinformatics tools rather than detailed experimental data. Only a few small experiments were described across the studies. Studies that focused on a single bioinformatics tool or primarily on CRISPR technology rather than the tools themselves were excluded. No data conversions were required, as there were very few data, and the purposes of the tools were so varied that comparisons would not have been meaningful.

Statistical techniques to explore heterogeneity were not employed. Few tools were mentioned across multiple studies, and when they were, their descriptions were highly consistent. As such, no further exploration of heterogeneity was deemed necessary.

### Appendix A.10. Reporting Bias Assessment

Given the nature of the studies, which primarily described tools, there were limited data to compare, and the risk of reporting bias was assessed qualitatively. Some studies described tools developed by the authors, potentially introducing bias due to overstatement of their own tools’ advantages. The risk of bias was not formally accessed, but studies were excluded primarily based on relevance, date, and quality, which indirectly reduced the possibility of biased reporting. Additionally, with only 50 studies screened out of over 2700 identified and many excluded early, the potential for missing or unpublished studies cannot be ruled out. However, no formal tools or tests (e.g., funnel plots) were used to assess reporting bias.

### Appendix A.11. Certainty Assessment

Certainty in the evidence was assessed qualitatively through careful reading of the articles, comparison of tool descriptions, and consideration of factors such as relevance, reliability, publication year, journal quality, citation counts, and consistency of results. The included studies showed minimal heterogeneity, and descriptions of tools were generally consistent across articles. Confidence in the evidence was based on subjective assessment rather than a specific framework (e.g., GRADE). However, potential bias was noted in studies where authors described their own tools.
